# Untargeted plasma metabolomics in canine cognitive dysfunction: the naturally occurring Alzheimer’s disease analog in dogs

**DOI:** 10.3389/fnins.2026.1681817

**Published:** 2026-03-17

**Authors:** Tonatiuh Melgarejo, Scarlett Harrison, Yan Chang, Miriam Munoz, Maya Kim, Youna Choi, Joyce G. Riveroll-Gonzalez, Barbara Natterson-Horowitz, Annika Linde

**Affiliations:** 1 College of Veterinary Medicine, Western University of Health Sciences, Pomona, CA, United States; 2 Modern Animal Hospital, Los Angeles, CA, United States; 3 College of Veterinary Medicine & Biomedical Sciences, Colorado State University, Fort Collins, CO, United States; 4 School of Veterinary Medicine, Ross University, Basseterre, Saint Kitts and Nevis; 5 Department of Global Health and Social Medicine, Harvard Medical School, Boston, MA, United States; 6 Department of Human Evolutionary Biology, Harvard University, Cambridge, MA, United States; 7 David Geffen School of Medicine, University of California, Los Angeles, Los Angeles, CA, United States

**Keywords:** aging, biomarkers, canine dementia, companion dogs, comparative neuropathology, lipid metabolism, translational model, untargeted metabolomics

## Abstract

**Introduction:**

Canine Cognitive Dysfunction (CCD) is an increasingly prevalent naturally occurring neurodegenerative condition in senescent dogs that share neuropathological and clinical features with human Alzheimer’s disease (AD). Metabolic profiling allows for identification of new candidates for AD biomarkers, diagnostics, and therapeutics. Despite its translational potential, plasma metabolomic profiling of dogs with CDD has not been previously characterized.

**Methods:**

This case–control study analyzed plasma samples from ten client-owned geriatric dogs, including five with severe CCD and five age-matched, clinically healthy controls. Untargeted plasma metabolomics was performed using ultra-performance liquid chromatography–mass spectrometry (UPLC–MS). Multivariate and univariate statistical analyses identified significant metabolic differences between the groups. Metabolites were considered significant based on a variable importance in projection (VIP) score > 1.5, fold change (FC) > 2.0, and adjusted *p*-value <0.05.

**Results:**

Fifteen metabolites across seven chemical classes were significantly altered in CCD dogs compared to controls, including glycerophospholipids, steroid derivatives, indoles, and mitochondrial-related compounds. Notably, elevated lysophosphatidic acid (LPA 20:2/0:0) and reduced ubiquinone-2 levels suggest dysregulation in neuroinflammatory and oxidative stress pathways. Cholesterol exhibited the highest FC and VIP scores, further reinforcing its role in AD pathogenesis. Hierarchical clustering and pathway enrichment analyses supported distinct metabolic signatures in CCD that mirror those observed in human AD.

**Discussion:**

This is the first untargeted plasma metabolomic profiling of dogs with CCD, revealing systemic metabolic disturbances that align with AD pathophysiology. Data was collected from senescent community-dwelling companion dogs, which enhances the study’s ecological and translational relevance. It supports the utility of CCD as an AD model and highlight candidate plasma biomarkers that warrant further investigation. Future longitudinal studies integrating metabolomics with neuroimaging, histopathology, and behavioral assessments are required to validate these findings and contribute to AD biomarker discovery and therapeutic development.

## 1 Introduction

Alzheimer’s disease (AD) represents the leading cause of dementia globally and is the fastest-growing epidemic (Sherzai et al., 2022; Zhang et al., 2024). Deaths from dementia are considered underreported, and some suggest AD may be the third leading cause of mortality in the US after cardiovascular disease and cancer (James et al., 2014). As a progressive neurodegenerative disorder marked by memory impairment, cognitive dysfunction, and behavioral changes, the pathology is characterized by accumulation of extracellular amyloid-beta plaques and intracellular neurofibrillary tangles composed of hyperphosphorylated tau protein. These changes are involved in driving synaptic dysfunction, neuronal loss, and progressive brain atrophy (Abubakar et al., 2022). The risk of developing dementia is significantly impacted by environmental factors, and a healthy lifestyle lowers the risk of AD substantially (Sherzai et al., 2022). However, despite decades of intensive research and major financial investments aimed at developing effective AD therapies, this pathology remains an incurable neurological disorder, which underscores the urgent need for innovative approaches to address this growing global health challenge (Sharma et al., 2025). Rodent models have contributed to an increased understanding of AD pathobiology; however, these laboratory models do not develop AD-like pathology naturally, and their translational utility is constrained by significant interspecies differences in brain architecture, immune function, and genetic composition. These disparities often result in findings that fail to accurately predict therapeutic outcomes in humans, thereby limiting the clinical relevance of rodent-based studies (Granzotto et al., 2024). The National Institute on Aging (NIA) has highlighted the critical need for alternative strategies to bridge this gap and pave a path towards successful AD drug development. Animal models that develop AD-like neurodegenerative conditions spontaneously are expected to provide more efficient and biologically relevant platforms for studying AD mechanisms and therapeutics. Still, the availability of naturally occurring animal models that faithfully recapitulate the complex pathology of AD is limited, which constitutes a barrier to translational research (Lamichhane et al., 2025). A few non-human animal species—including dog, degu, rhesus macaque, green monkey, and marmoset—are viewed as potential alternative models given their propensity for spontaneous AD-like neurodegenerative disease (Steffen et al., 2016; Zeiss, 2020). The dog stands out as the only domesticated species, and companion dogs constitute a unique model system for more relevant and ethical AD research because they live in human households with shared environmental exposures, unlike laboratory Beagles.

Canine cognitive dysfunction (CCD) is a naturally occurring neurodegenerative condition in geriatric dogs that is considered the analog of human AD (Dewey et al., 2019; Head, 2013). Aging dogs with CCD have been recognized as a unique and valuable animal model for AD research for over two decades due to spontaneous isomorphic cellular changes that closely mirror human brain pathology and behavioral deficits (Adams et al., 2000; Head et al., 2001; Mihevc and Gregor, 2019). However, the dog model is considered expensive and technically challenging, which has limited its widespread adoption in AD research. This underutilization stems primarily from higher husbandry costs, long-term housing requirements, complexity of rigorous behavioral evaluations (Bosch et al., 2012), and owner compliance challenges when clients euthanize dogs with CCD prematurely due to declining quality of life. Despite these practical barriers, the limitations of traditional laboratory animal models pose a significant obstacle to developing effective disease-modifying therapies for AD (Lamichhane et al., 2025). With both AD (Livingston et al., 2024) and CCD (Dewey et al., 2019) rising in prevalence due to aging populations and improved diagnostic awareness, there is a compelling rationale to re-evaluate companion dogs with spontaneous CCD (Schütt et al., 2018) as a translational, non-transgenic animal model for AD research.

Untargeted metabolomics is a powerful analytical approach that enables comprehensive profiling of small-molecule metabolites in biological samples and offers insights into systemic physiological and pathological states. When applied to minimally invasive biofluids such as plasma, this technique facilitates the identification of metabolic alterations associated with health and disease. The aim of this study was to characterize the untargeted plasma metabolomics profiles in a cohort of dogs with spontaneous CCD compared to controls. The research sought to advance comparative investigations into metabolic events implicated in the neurodegenerative processes associated with dementia in dogs of predicted future translational value for human AD research.

## 2 Methods

### 2.1 Study design and aim

This is a single-center, case–control study aiming to comprehensively analyze plasma metabolomic profiles of client-owned, community-dwelling, companion dogs diagnosed with canine cognitive dysfunction (CCD) compared to age-matched clinically healthy controls.

### 2.2 Animals

A total of 10 companion dogs older than 10 years were included in the study with prior client consent. The CCD group (*n* = 5) included dogs with a previously confirmed diagnosis of Canine Cognitive Dysfunction, as determined by a licensed veterinarian based on clinical signs and a canine dementia scale (CADES) score above 44 points (Madari et al., 2015). The control group (*n* = 5) included age-matched clinically healthy companion dogs with a CADES score below 8 points. Exclusion criteria included significant hematologic or serum chemistry abnormalities, other significant comorbidities, non-CCD neuropathology, psychopharmacological therapy, behavioral problems unrelated to CCD, and age younger than 10 years.

We selected 10 years as the age cut-off because cognitive decline and neuropathological changes consistent with Canine Cognitive Dysfunction (CCD) are most observed in dogs older than 10 years, regardless of breed. While aging rates vary by breed, Dewey et al. (2019) emphasize that CCD prevalence increases markedly above this threshold across breeds, making it a practical criterion for geriatric classification in clinical studies.

### 2.3 Hematology and serum chemistry analysis

Blood (6 mL) was collected via cephalic or jugular venipuncture for a complete blood count (CBC), serum chemistry panel (SCP), and untargeted plasma metabolomics (UPM) analyses. The sample volume was divided evenly (2 mL per tube) into a red cap-yellow ring (serum clot activator), purple cap (EDTA), and green cap (lithium heparin) tubes, used for CBC, SCP, and UPM analyses, respectively. The CBC and SCP analyses were performed at the WesternU Pet Health Center using a ProCyte Dx Hematology Analyzer (CBC), and Catalyst One Chemistry Analyzer (SCP), both from IDEXX Laboratories (Westbrook, ME). Data was analyzed using MS Excel for Mac (Version 16.97.2) for descriptive and inferential statistics (*t*-test and alpha level of 0.05).

### 2.4 Sample preparation for untargeted plasma metabolomics

Whole blood samples for UPM (2 mL in lithium heparin tubes) were centrifuged at 3,000 rpm x 10 min and 1 mL of plasma was collected from each sample, stored at −80 °C and batched prior to overnight shipping on dry ice to Creative Proteomics (CP) (Shirley, NY) for untargeted plasma metabolomics analysis. The following protocol was provided by CP. Briefly, samples were thawed on ice, and 60 μL of each sample was subsequently transferred into a tube containing 1.5 mL of chloroform: methanol (2:1, v/v) and 0.5 mL of ultrapure water. The mixture was homogenized for 90 s., vortexed for 1 min, and sonicated for 30 min at 4 °C. Samples were then centrifuged at 3,000 rpm for 10 min at 4 °C, and the lower phase was transferred to a new tube and dried under nitrogen gas. The dried extract was resuspended in 200 μL isopropyl alcohol: methanol (1:1, v/v), followed by the addition of 5 μL LPC (12:0) as an internal standard. After centrifugation at 12,000 rpm for 10 min at 4 °C, the supernatant was collected for liquid chromatography–mass spectrometry (LC–MS) analysis. Quality control (QC) samples were prepared by pooling equal amounts of extract from each sample, following the same sample preparation procedure. QC samples were included in the analysis to assess the stability and reproducibility of the method.

### 2.5 Ultra-performance liquid chromatography-mass spectrometry

Metabolite separation was performed by CP using ultra-performance liquid chromatography (UPLC) coupled with a Q Exactive mass spectrometer (Thermo Fisher Scientific, Waltham, MA). Chromatographic separation was achieved using an ACQUITY UPLC BEH C18 column (100 × 2.1 mm, 1.7 μm). The mobile phase consisted of solvent A (60% ACN + 40% H2O + 10 mM HCOONH4) and solvent B (10% ACN + 90% isopropyl alcohol+10 mM HCOONH4). A gradient elution program was applied, starting with 30% B from 0 to 1.0 min, increasing to 100% B from 1.0 to 10.5 min, maintaining at 100% B from 10.5 to 12.5 min, and returning to 30% B from 12.5 to 12.51 min, where it remained until 16 min. The flow rate was set at 0.3 mL/min, with the column temperature maintained at 40 °C and the sample manager temperature at 4 °C. For mass spectrometry, both electrospray ionization positive (ESI+) and negative (ESI−) modes were utilized. In ESI + mode, the heater temperature was 300 °C, sheath gas flow rate was set at 45 arbitrary units (arb), auxiliary gas flow rate at 15 arb, sweep gas flow rate at 1 arb, spray voltage at 3.0 kV, capillary temperature at 350 °C, and S-Lens RF level at 30%. In ESI − mode, similar parameters were applied, except for the spray voltage, which was set at 3.2 kV, and the S-Lens RF level, which was set at 60%.

### 2.6 Statistical analysis and metabolite identification

All study samples (*n* = 10) were analyzed in a single analytical batch to eliminate inter-batch variability. Quality control (QC) samples were injected periodically throughout the analytical sequence to monitor instrument performance and system stability. Following data acquisition, peak detection and alignment were performed using Compound Discoverer 3.0 (Thermo Fisher Scientific), with metabolite peak areas from ESI + and ESI − modes merged for statistical analysis. Missing values were imputed using half the minimum detected value for each metabolite to facilitate statistical analysis while minimizing bias from low-abundance features. Data normalization was performed using total ion normalization, where the peak area of each metabolite was divided by the sum of all detected metabolite peak areas within the same sample and multiplied by 1,000,000 to generate values on a parts per million (ppm) scale. This approach corrects for variation in overall ion intensity between samples. The normalized dataset was then autoscaled using unit variance (UV) scaling prior to multivariate statistical analysis. QC samples were injected throughout the batch to monitor analytical stability; QC1 was the first injection in the run but was not used for normalization. Post normalization assessment confirmed that all study samples fell within the 95% confidence interval, indicating stable analytical performance. The relative standard deviation (RSD) distribution of QC samples showed that most detected features had RSD values below 30%, indicating acceptable analytical reproducibility. Prior to multivariate analysis, normalized metabolite abundance values were log-transformed and median-centered to reduce the influence of highly abundant features and improve data distribution. Principal Component Analysis (PCA) was performed on preprocessed data using SIMCA-P (version 14.1, Umetrics) as an unsupervised method to visualize overall metabolic patterns. Variable Importance in Projection (VIP) scores, fold-change (FC) values, and *t*-test *p*-values with Benjamini-Hochberg false discovery rate (FDR) correction were calculated to identify significantly altered metabolites. Metabolites were considered significant if they met the following criteria: VIP score >1.5, FC > 2.0, and Benjamini-Hochberg-adjusted *p*-value <0.05. Hierarchical clustering analysis (HCA) was performed using the complete linkage algorithm in Cluster 3.0 (Stanford University), with visualization carried out using Seaborn’s clustermap function. Metabolite identification was performed using MS/MS fragmentation pattern matching against the Human Metabolome Database (HMDB) and LipidSearch spectral libraries, with identifications reported at Metabolomics Standards Initiative (MSI) confidence level 2, indicating putative annotation based on accurate mass, retention time, and MS/MS spectral similarity to reference compounds. MSI level 2 identifications represent high-confidence structural assignments without confirmation by authentic chemical standards. Where available, additional confidence was obtained by comparing fragmentation patterns with published literature on the specific metabolite class. Pathway analysis was performed using KEGG and MetaboAnalyst 5.0 to determine the metabolic pathways most affected in CCD.

## 3 Results

### 3.1 Study subjects and groups

Dogs enrolled in the study were client-owned, neutered (spayed females, castrated males), with a median age of 13 years (range 10–16 yrs); 40% were male. Breeds included Australian shepherd, boxer, dachshund, German shorthaired pointer, Labrador retriever mix (2), and Chihuahua (4). Signalment and health status of all study subjects are included in Supplementary Table S1. For the CCD group, dogs were confirmed to have severe cognitive impairment based on clinical signs and a CADES score ≥45. Control dogs had a CADES score ≤7 (Supplementary Table S2).

### 3.2 Complete blood count and serum chemistry panel

Comprehensive hematological (CBC) and biochemical (SCP) profiling revealed neither clinically relevant abnormalities in either group nor statistically significant differences between the groups (Supplementary Table S3).

### 3.3 Plasma untargeted metabolomics

#### 3.3.1 Quality control assessment

To ensure the stability of the LC-MS system, QC samples were analyzed at regular intervals in both positive and negative ion modes. The relative standard deviation (RSD) distribution, shown in Supplementary Figure S1, indicated that most RSD values were below 30%, confirming the robustness of the analytical method.

#### 3.3.2 Multivariate statistical analysis

**Figure 1:**
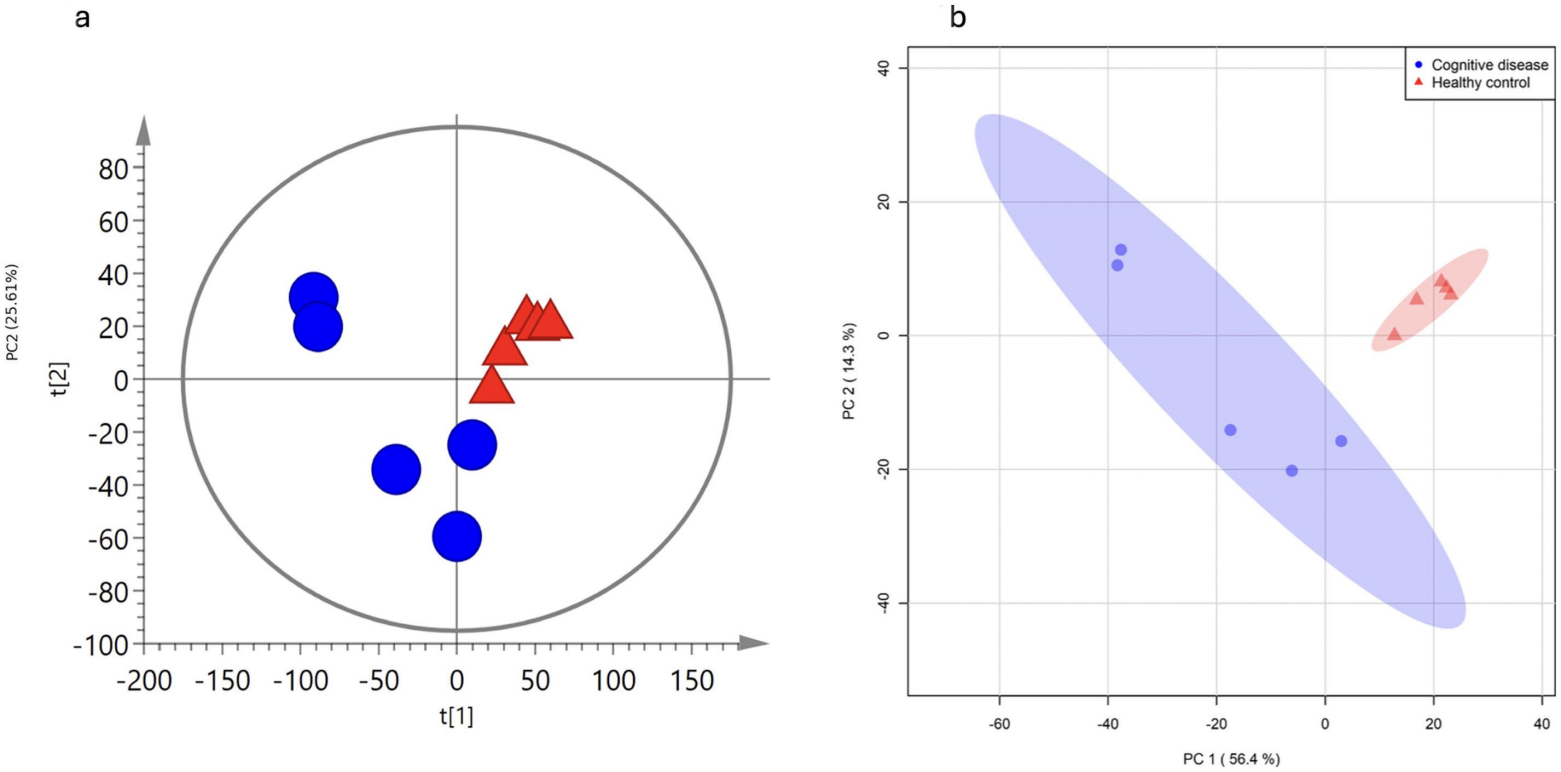
Principal component analysis (PCA) score plots demonstrating metabolic variation between dogs with canine cognitive dysfunction (CCD) (blue circles) and clinically healthy controls (CH) (red triangles). **(a)** Two-dimensional PCA score plot showing sample distribution based on the first two principal components. Each point represents an individual dog (*n* = 5 per group), and ellipses denote 95% confidence intervals. The spatial separation between groups indicates distinct metabolic phenotypes, with some degree of overlap reflecting biological variability within this small cohort. **(b)** PCA score plot generated using alternative visualization parameters, confirming the metabolic clustering pattern observed in **(a)**. PC1 accounts for 56.4% of total variance while PC2 explains 14.3%, together capturing 70.7% of metabolic variation. The unsupervised nature of PCA analysis reveals inherent metabolic differences between CCD and control groups without imposing class labels, providing an unbiased overview of the dataset structure and supporting subsequent supervised discriminant analyses for biomarker identification.

Normalization was performed to ensure all samples were within a 95% confidence interval, confirming system stability. PCA analysis revealed clear clustering patterns, reflecting distinct metabolic variations between groups (Figures 1a,b). Model validation metrics, including R2(Y) goodness-of-fit, Q2(Y) predictive reliability, and permutation testing, were not performed as part of the initial analytical workflow. Given the small sample size (*n* = 5 per group) and exploratory study design, we emphasize unsupervised principal component analysis (PCA) and stringent univariate selection criteria (VIP > 1.5, FC > 2.0, FDR-adjusted *p* < 0.05) rather than relying exclusively on supervised multivariate classification for biomarker identification.

#### 3.3.3 Single variable analysis

**Figure 2:**
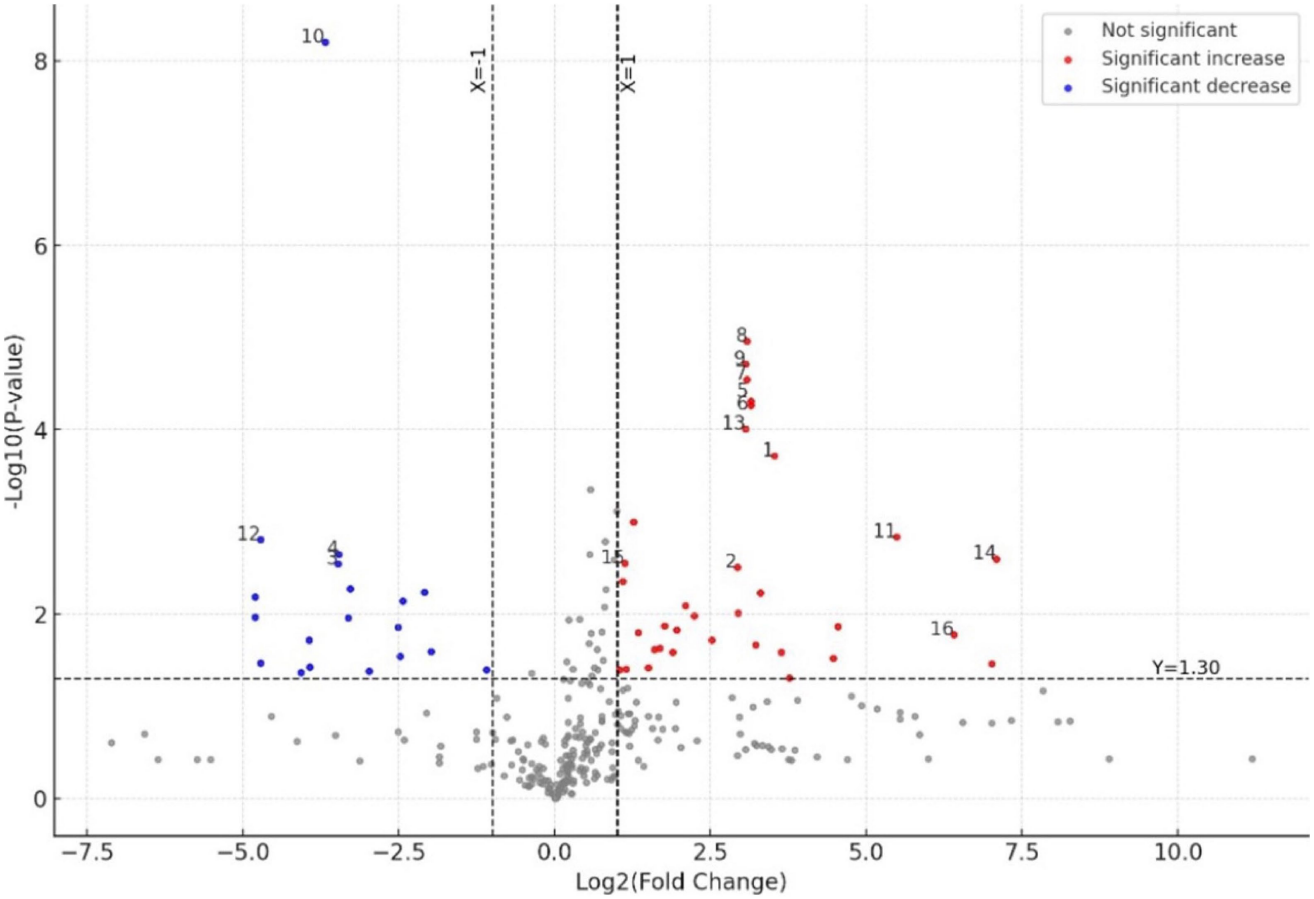
Volcano plot illustrating differential metabolite expression between the canine cognitive dysfunction (CCD) group vs. the control group. The *x*-axis represents the log₂(fold change), and the *y*-axis shows the –log₁₀(*p*-value). The horizontal dashed line corresponds to the significance threshold (FDR-adjusted *p*-value < 0.05; −log₁₀(*p*) ≈ 1.30), and the vertical dashed lines indicate the fold change cutoffs (log₂FC = ±1). Metabolites significantly upregulated in the CCD group are shown in red, while those significantly downregulated are shown in blue. Metabolites that did not meet the significance threshold are shown in gray. Select metabolites of specific interest are annotated by number as follows: 1: Prop-2-enylcyclohexane; 2: 4,6,8-Megastigmatriene; 3: Indoleacetaldehyde; 4: Boschniakine; 5: 2-4-Dodecadienal; 6: 2-Cyclotetradecen-1; 7: Humulol; 8: Arctiol; 9: 8-Isoprostane; 10: Ubiquinone-2; 11: Cholesta-3,5-diene; 12: PC(18:0/14:1); 13: 5-Heneicosyl-1,3-benzenediol; 14: Fecosterol; 15: LysoPA(20:2/0:0); 16: Cholesterol.

Metabolites were selected as significant if they met the criteria of VIP > 1.5, FC > 2.0, and an adjusted *p*-value below 0.05 (Table 1). Using these criteria, fifteen AD-associated metabolites were identified, including glycerophospholipids, indoles, polyprenylbenzoquinones, pyridines, saturated hydrocarbons, and steroid derivatives. Cholesterol, although not meeting the adjusted *p*-value threshold (*p* = 0.11), was included as a biologically relevant metabolite due to having the highest FC and VIP among all significant plasma metabolites, combined with existing evidence linking elevated cholesterol levels to amyloid-beta accumulation, neuroinflammation, and vascular contributions to cognitive impairment (Livingston et al., 2024). The volcano plot in Figure 2 highlights key metabolic differences between the groups (CCD vs. CH).

**Table 1:** Select plasma metabolites in dogs with canine cognitive dysfunction (CCD) vs. clinically healthy controls (CH).

Class	Formula	AVE (CCD)	AVE (CH)	FC (CCD/CH)	Adj. *p*-value	VIP	AD biomarker
Glycerophospholipids							
LysoPA(20:2/0:0)	C23H43O7P	116.61	53.63	2.17	0.04	1.66	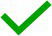
PC(18:0/14:1)	C40H78NO8P	13.05	345.13	0.04	0.03	1.75	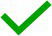
Indoles and derivatives							
Indoleacetaldehyde	C10H9NO	6.54	72.66	0.09	0.04	1.53	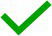
Organooxygen compounds							
2-4-Dodecadienal	C12H20O	44.16	5.00	8.83	0.002	1.69	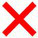
2-Cyclotetradecen-1	C14H24O	37.21	4.20	8.87	0.003	1.69	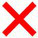
Phenols							
5-Heneicosyl-1,3-benzenediol	C27H48O2	7.54	0.90	8.33	0.004	1.64	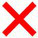
Polyprenylbenzoquinones							
Ubiquinone-2	C19H26O4	11.64	149.93	0.08	<0.0001	1.66	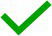
Prenol lipids: sesquiterpenoids							
Humulol	C15H26O	32.97	3.90	8.46	0.002	1.69	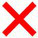
Arctiol	C16H28O	30.77	3.63	8.48	0.002	1.68	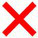
Iridoid alkaloids							
Boschniakine	C10H11NO	53.97	597.95	0.09	0.04	1.51	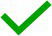
Saturated hydrocarbons							
8-Isoprostane	C20H40	70.69	8.48	8.34	0.002	1.69	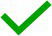
Steroids and steroid derivatives							
Cholesta-3,5-diene	C27H44	89.85	2.01	44.77	0.03	1.65	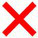
Cholesterol	C27H46O	16.25	0.19	84.96	0.11	1.74	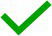
Fecosterol	C28H46O	32.85	0.24	135.86	0.04	1.74	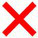
Unsaturated hydrocarbons							
Prop-2-enylcyclohexane	C9H16	43.64	3.80	11.49	0.007	1.72	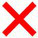
4,6,8-Megastigmatriene	C13H20	54.08	7.12	7.60	0.04	1.66	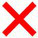

AD, Alzheimer’s disease; AVE, average (metabolite abundance); FC, fold change; VIP, variable importance in projection. The last column indicates whether (green checkmark) or not (red cross) a metabolite is a related biomarker for Alzheimer’s disease.

#### 3.3.4 Cluster analysis

**Figure 3:**
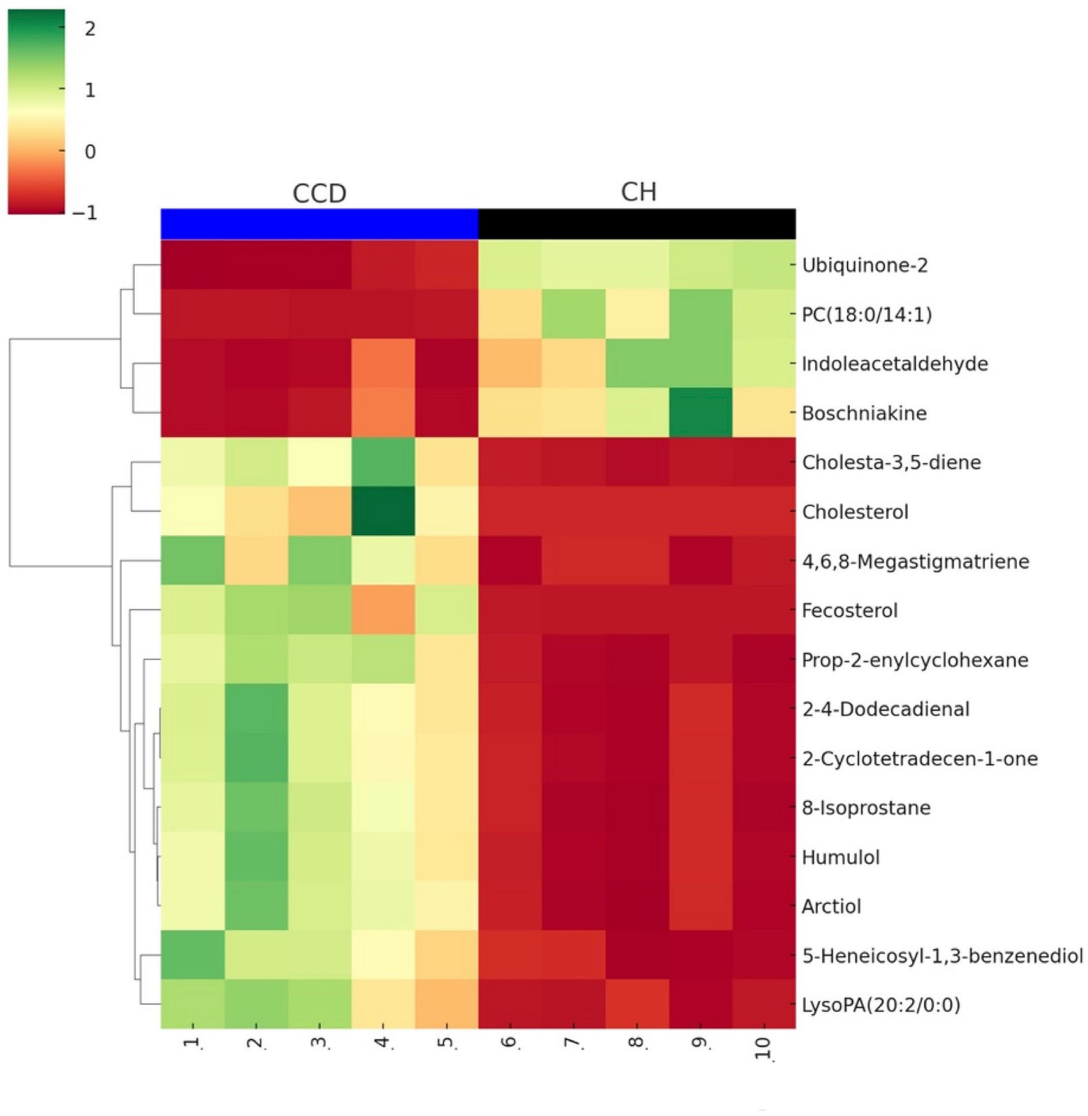
Heatmap illustrating the distribution of select metabolites across the canine cognitive dysfunction (CCD) vs. clinically healthy control (CH) groups. Each row represents a metabolite, and each column corresponds to an individual sample (CCD: 1–5, CH: 6–10). Color intensity reflects relative abundance, with green indicating increased levels and red indicating decreased levels relative to the median for each metabolite. Hierarchical clustering was applied to group metabolites based on similarity in expression patterns.

To identify trends in metabolite variation, mean metabolite content from the groups was used to calculate metabolite ratios. After log transformation and normalization, hierarchical clustering analysis (HCA) was performed. The heatmap in Figure 3 illustrates the distribution of significant metabolites (plus cholesterol) in dogs with CCD vs. CH (controls), where red represents increased levels and green indicates decreased levels relative to the median.

#### 3.3.5 Metabolite correlation network

**Figure 4:**
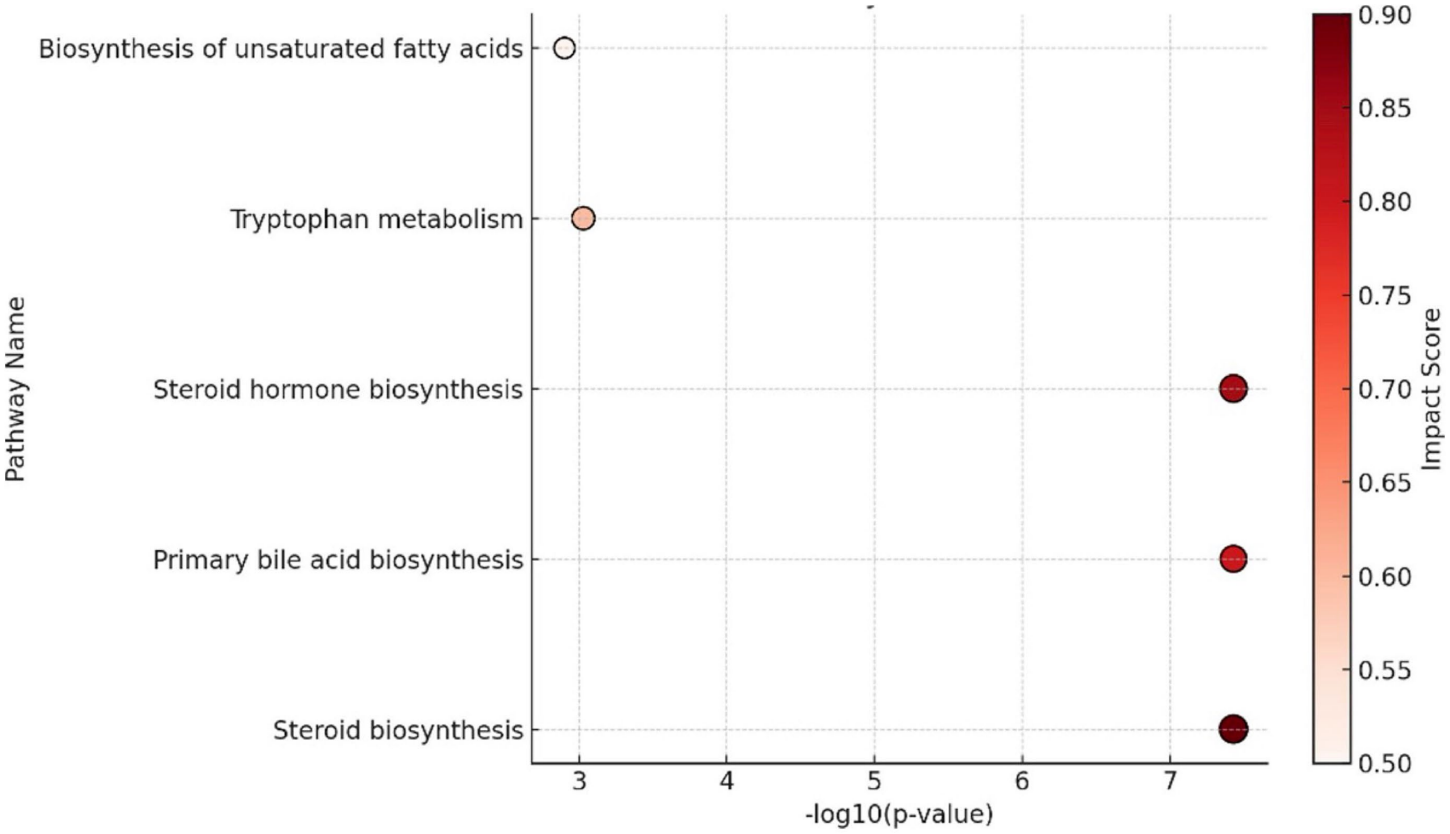
Pathway enrichment and topology analysis of perturbed metabolites between the canine cognitive dysfunction (CCD) and the clinically healthy control (CH) groups. The dot plot displays the top enriched metabolic pathways, with the *x*-axis representing pathway significance (−log₁₀(*p*-value)) and the color intensity indicating the pathway impact score, based on pathway topology analysis. Larger and darker red circles represent pathways with higher impact scores and greater biological relevance. Steroid biosynthesis, primary bile acid biosynthesis, and steroid hormone biosynthesis were among the most significantly enriched and impactful pathways.

A metabolome view (Figure 4) was generated based on pathway enrichment and topology analysis. Each node in the network represents a metabolite set, with its size indicating fold enrichment and its color intensity indicating statistical significance. Metabolite sets were considered connected when they shared more than 25% of their combined metabolites; the color gradient ranged from pink to red, indicating increasing significance.

## 4 Discussion

This study provides a comprehensive analysis of plasma metabolomic profiles in companion dogs diagnosed with severe CCD compared to age-matched clinically healthy controls. Utilizing an untargeted metabolomic approach, our investigation revealed significant alterations in multiple plasma metabolite classes in the CCD group that mirror those associated with AD in people, including glycerophospholipids, indoles, polyprenylbenzoquinones, iridoid alkaloids, saturated hydrocarbons, and steroid derivatives. Our data revealed significant differences in plasma glycerophospholipid concentrations between the groups (CCD vs. CH). Specifically, there was a marked increase in lysophosphatidic acid (LPA) 20:2/0:0 in CCD dogs compared to controls. LPA 20:2/0:0, a bioactive lipid, plays a crucial role in cellular signaling processes. LPAs have been implicated in several key pathological mechanisms in AD (Ahmad et al., 2024). Notably, LPAs activate microglia and astrocytes, leading to the release of pro-inflammatory cytokines that significantly contribute to the progression of AD through neuroinflammation (Hao et al., 2020). LPAs enhance amyloid-beta (Aβ) production by upregulating beta-secretase expression, which is crucial for the formation of amyloid plaques, a hallmark of both AD and CCD (Hao et al., 2020; Head et al., 2001). Additionally, LPAs exacerbate neurodegeneration by compromising the integrity of the blood–brain barrier, allowing harmful substances to enter the brain. LPAs are also involved in phosphorylating tau proteins, leading to the formation of neurofibrillary tangles, another key feature of AD pathology (Hao et al., 2020). We detected elevated LPA 20:2/0:0 plasma concentration in the CCD group, which is relevant as recent studies have shown that aged canines with CCD exhibit phosphorylated tau at threonine 217 (Hines et al., 2023). The study also showed that PC 18:0/14:1 was significantly lower in the CCD group, a plasma glycerophospholipid essential for maintaining cellular membrane integrity and fluidity. This finding runs parallel to metabolomics data in people with AD. The reduction in PC 18:0/14:1 appears to have biological effects similar to the increase in LPA 20:2/0:0 in humans with AD, namely, enhancing neuroinflammation (Lee and Chang, 2025), amyloid-beta (Aβ) aggregation (Kurz et al., 2025), and exacerbating tau hyperphosphorylation (Kurz et al., 2025). Therefore, plasma glycerophospholipid alterations in dogs with CCD appear to be a crucial component of neurodegenerative processes in the canine brain and should be considered a comparative biomarker to further enhance our understanding of AD pathophysiology.

In this study, the plasma concentration of ubiquinone-2, a key component of the mitochondrial electron transport chain, was significantly reduced in the CCD group compared to controls. The role of the coenzyme Q class of molecules (including ubiquinone-2 and CoQ10) in the pathogenesis of AD has been investigated, primarily focusing on their involvement in abnormal mitochondrial metabolism and oxidative stress (Fišar and Hroudová, 2024). The decrease in ubiquinone-2 in dogs with CCD most likely reflects mitochondrial dysfunction, a hallmark of AD pathogenesis (Ashleigh et al., 2023; Wang et al., 2020). Oxidative stress is one of the primary mechanisms involved in the pathogenesis of AD and other neurodegenerative disorders. In humans with AD, systematic reviews have shown that serum and plasma CoQ10 levels do not differ significantly between patients and controls, suggesting that peripheral measurements may not accurately reflect brain tissue deficits (Jiménez-Jiménez et al., 2023). Moreover, studies examining CoQ10 supplementation in humans with AD and mild cognitive impairment (MCI) have yielded inconclusive results (Bagheri et al., 2023). Despite these equivocal findings in human observational and interventional studies, experimental rodent models of AD have demonstrated that CoQ10 supplementation confers neuroprotective benefits and improves memory function (Dumont et al., 2011). Our observation of significantly reduced plasma ubiquinone-2 in dogs with CCD presents a potentially important distinction: this measurable peripheral biomarker may better reflect central nervous system pathology in the naturally occurring canine model than analogous measurements do in humans. The discrepancy between inconclusive findings in human studies and positive therapeutic outcomes in rodent models underscores the translational value of investigating CoQ10 in companion dogs with spontaneous CCD. In this context, dogs may serve as a critical bridge species where peripheral biomarkers more reliably predict therapeutic response, thereby informing clinical trial design and treatment strategies for human AD. Future studies examining CoQ10 supplementation in dogs with CCD could therefore provide valuable insights into whether this intervention merits renewed investigation in human patients, particularly if canine plasma levels are mechanistically linked to cognitive outcomes.

Interestingly, we observed a substantially higher plasma concentration of indole-acetaldehyde in the clinically healthy controls than in the CCD group. Indole derivatives, including indoleacetaldehyde, have been investigated for their ability to inhibit monoamine oxidase (MAO) and cholinesterase, enzymes involved in the breakdown of neurotransmitters. Inhibition of these enzymes can help reduce oxidative stress and neuroinflammation (Denya et al., 2018). Studies have demonstrated that certain indole derivatives, including those with indoleacetaldehyde moieties, exhibit significant neuroprotective effects against neurotoxic insults in cellular models. These compounds show improved chemical stability, making them promising candidates for AD therapeutics development (Ciaglia et al., 2024). Indole derivatives are primarily produced in the human body through metabolism of tryptophan by intestinal microorganisms (Li et al., 2021). The gut microbiota plays a crucial role in converting tryptophan into indole derivatives that are released in the plasma where they can exert a variety of biological effects. The reduction in indoleacetaldehyde plasma concentration in the CCD group points to alterations in tryptophan metabolism, which has also been associated with neuroinflammation and neurodegeneration in AD (Xie et al., 2024). Since indole derivatives can influence gut-brain axis signaling, their dysregulation in CCD may reflect similar pathways operative in AD, thus emphasizing the translational relevance.

We found that plasma boschniakine, an iridoid alkaloid from the plant *Boschniakia rossica* (BR), was ten times higher in controls compared to the CCD group. BR contains more than 100 chemical constituents, most prominently boschnaside, boschniakine, 7-deoxy 8-epiloganic acid, and (4R)-4-hydroxymethyl-boschnialactone, which possess antioxidant and anti-inflammatory properties (Fan and Ren, 2019). Recent studies in rodent models of AD demonstrated that BR significantly increases hippocampal neuronal populations and improves learning and memory (Hu et al., 2025). None of the dogs in our study received herbal supplements or botanical products, as verified during enrollment. We hypothesize that the source of boschniakine may be plant ingredients commonly used in commercial dog food manufacturing, such as blueberry or white willow, though BR itself is an obligate parasitic plant native to the Northern Hemisphere and does not grow south of the Alaska Panhandle (Konishi et al., 1987). However, we acknowledge that accurate metabolite identification is an inherent challenge in untargeted metabolomics, and the possibility of structural misidentification or detection of an isomeric compound cannot be entirely excluded. The signal was detected across samples in both groups, with marked differential abundance. While our dietary hypothesis provides a plausible explanation, the true origin of this compound and its biological relevance in CCD warrant further investigation through targeted analytical methods and dietary analysis. Nevertheless, the convergence of our findings with experimental evidence from rodent AD models suggests that this metabolite class merits continued attention in comparative dementia research.

F2-isoprostanes, including the isomer 8-isoprostane, are products of non-enzymatic peroxidation of arachidonic acid, a polyunsaturated fatty acid in cell membranes. Growing evidence links oxidative stress and lipid peroxidation to the pathogenesis of neurodegenerative diseases such as AD (Miller et al., 2014). Elevated levels of F2-isoprostanes have been observed in neurons, cerebrospinal fluid, plasma, and urine of individuals with AD and MCI, indicating oxidative damage in the brain (Casadesus et al., 2007; Praticò et al., 2002). In this study, the CCD group had a significantly higher plasma 8-isoprostane level, suggesting increased brain oxidative stress. While the causal relationship between F2-isoprostane elevation and AD remains under investigation (Trares et al., 2022), these findings have translational relevance. Companion dogs represent a unique spontaneous animal model that mirrors the pathways implicated in human AD, where oxidative damage is a key contributor to neurodegeneration (Praticò, 2010).

Cholesterol regulation in the brain involves complex biochemical pathways. Steroid derivatives, such as cholesterol, are essential for neural health, particularly for synapse formation and signal transmission. Disruptions in cholesterol metabolism are linked to neurodegeneration and are considered key contributors to AD pathogenesis, especially in late-onset cases where major genetic risk factors are associated with cholesterol regulation (Dai et al., 2021; Feringa and van der Kant, 2021). This underscores a need for further investigation into cholesterol’s role in neurodegenerative diseases. In our study, the CCD group showed the second-highest plasma cholesterol levels of FC and VIP among the selected plasma metabolites (Table 1). While the mechanisms remain unclear, cholesterol is known to influence amyloid precursor protein processing and Aβ accumulation in the human brain (Dai et al., 2021), suggesting that similar pathways may exist in dogs with CCD. In fact, the Aβ peptide family includes AD-associated peptides with identical amino acid sequences between dogs and humans (Head et al., 2001). Notably, Aβ42 deposition in three brain areas correlated strongly with canine cognitive dysfunction scale (CCDS) scores (Urfer et al., 2021), supporting the possible link between altered cholesterol metabolism and Aβ42 pathology in this species.

Our untargeted metabolomic approach complements recent targeted proteomic studies in canine cognitive decline. Alsulami et al. (2025) demonstrated that established human AD biomarkers, including Aβ40, Aβ42, p-tau181, NfL, and GFAP, are detectable in canine plasma and show significant alterations in dogs with cognitive dysfunction, providing important validation of the translational relevance of protein biomarkers in this model. While their targeted approach confirmed the presence of known AD-associated proteins, our discovery-based metabolomic profiling identified novel alterations in lipid metabolism, mitochondrial function, and oxidative stress pathways that may represent complementary or upstream pathogenic mechanisms. The convergence of proteomic and metabolomic evidence strengthens the case for CCD as a faithful analog of human AD. Future studies integrating both approaches, combining targeted quantification of established protein biomarkers (Aβ40, Aβ42, NfL, GFAP, p-tau217) with comprehensive metabolic profiling, will be essential to develop multi-modal biomarker panels that capture the full complexity of neurodegenerative processes in this naturally occurring model. Such integrative approaches may ultimately identify biomarker combinations with superior diagnostic and prognostic performance for both canine and human dementia.

Nine additional circulating metabolites showed significant differences in plasma concentrations between groups, representing potentially novel biomarkers for CCD that warrant detailed consideration. Among the steroid derivatives, fecosterol (FC = 135.86) exhibited the highest fold-change of any metabolite in our analysis. Fecosterol is a phytosterol and intermediate in cholesterol biosynthesis that has been implicated in cellular membrane dynamics and lipid raft organization (Shrivastava et al., 2023). Disrupted sterol metabolism has been increasingly recognized as a feature of neurodegenerative disease, with alterations in membrane lipid composition affecting amyloid precursor protein processing and neuronal signaling (Shin et al., 2024). The dramatic elevation of fecosterol in CCD dogs suggests profound disturbances in sterol homeostasis that may parallel cholesterol dysregulation observed in human AD. Two sesquiterpenoids: humulol (FC = 8.46) and arctiol (FC = 8.48) were similarly elevated in the CCD group. Sesquiterpenoids are volatile lipophilic compounds derived from the mevalonate pathway that possess antioxidant and anti-inflammatory properties in plant systems (Yang et al., 2018). While their endogenous roles in mammalian metabolism remain poorly characterized, elevated levels may reflect compensatory antioxidant responses to oxidative stress or, alternatively, altered metabolism of dietary terpenes. Interestingly, certain sesquiterpenoids have demonstrated neuroprotective effects in cellular models of neurodegeneration by modulating inflammatory signaling and reducing oxidative damage (Singh and Singh, 2025), suggesting that these compounds may represent adaptive responses to neurodegenerative processes. Among organooxygen compounds, both 2,4-dodecadienal (FC = 8.83) and 2-cyclotetradecen-1-one (FC = 8.87) were markedly elevated in CCD dogs. These *α*,*β*-unsaturated aldehydes are products of lipid peroxidation and are established markers of oxidative stress (Lee and Park, 2013). Their accumulation in CCD plasma aligns with our observation of elevated 8-isoprostane and suggests that oxidative damage to membrane lipids is a prominent systemic feature of canine cognitive dysfunction. Reactive aldehydes can form protein adducts, impair cellular function, and propagate oxidative cascades, contributing directly to neuronal injury (Pizzimenti et al., 2013). The phenolic compound 5-heneicosyl-1,3-benzenediol (FC = 8.33) and unsaturated hydrocarbons, including prop-2-enylcyclohexane (FC = 11.49) and 4,6,8-megastigmatriene (FC = 7.60), were also significantly elevated in CCD. While the biological roles of these specific compounds in neurodegenerative disease are not yet established, their chemical structures suggest involvement in lipid signaling and membrane dynamics. Collectively, these metabolic shifts indicate broad disruptions affecting energy homeostasis, membrane function, oxidative balance, and cellular signaling pathways. The convergence of multiple independent markers of oxidative stress, lipid peroxidation, and sterol dysregulation reinforces the systemic nature of metabolic dysfunction in CCD and strengthens the biochemical parallels between canine cognitive dysfunction and human Alzheimer’s disease.

This study analyzed peripheral plasma exclusively, without CSF biomarkers, neuroimaging, or neuropathological validation. Our mechanistic interpretations regarding CNS pathology represent hypothesis-generating associations based on literature parallels rather than direct evidence. Future studies integrating metabolomics with CSF biomarkers and neuropathology are essential to establish causative relationships.

The metabolic alterations identified in this study become more urgent when considered in light of the current therapeutic landscape. Despite decades of research investment, treatment options for both AD and CCD remain limited and largely symptomatic. Recently approved monoclonal antibodies for clinical management of AD, including lecanemab and donanemab, target amyloid-*β* pathology and demonstrate modest slowing of cognitive decline in early-stage disease, yet offer limited clinical benefit, carry significant adverse effects, including amyloid-related imaging abnormalities (ARIA), and do not reverse neurodegeneration (Livingston et al., 2024). For CCD, selegiline (Anipryl), a monoamine oxidase-B inhibitor, remains the only FDA-approved pharmaceutical therapy, though clinical responses are variable and often modest (Dewey et al., 2019). Adjunctive neuroprotective supplements and environmental enrichment strategies show inconsistent efficacy, with many dogs demonstrating minimal sustained improvement in moderate to severe disease. This therapeutic void in both species underscores the critical need for biomarker-driven approaches that enable earlier diagnosis, improve patient stratification for clinical trials, and identify novel disease-modifying targets; precisely the translational objectives our plasma metabolomic profiling aims to advance.

### 4.1 Strengths and limitations

The total sample size of this study is a clear limitation, but it reflects the exploratory, hypothesis-generating nature of this pilot work and the constraints inherent to naturally occurring clinical populations rather than diminished methodological rigor. Comparable small-cohort designs have been used in veterinary metabolomics, including an untargeted LC–MS study of atypical myopathy in horses that analyzed 5 affected and 11 healthy animals (Wouters et al., 2021). This context underscores the need for larger follow-up studies while situating our approach within established precedent. We acknowledge that, with this limited cohort, the risk of false-positive discoveries is elevated, particularly because untargeted metabolomics typically generates hundreds to thousands of metabolite features for analysis. To mitigate this risk, we applied stringent statistical criteria for biomarker selection, including FDR-adjusted *p*-values (*p* < 0.05), VIP scores>1.5, and fold changes>2.0. These conservative thresholds were deliberately chosen to minimize false discoveries while recognizing that independent validation in larger cohorts remains essential. The pilot study is limited by considerable demographic and lifestyle heterogeneity among enrolled dogs, including variability in breed, body size, sex, neuter status, and unrecorded factors such as diet, activity, and environmental enrichment. These variables may independently influence metabolic profiles, but were not controlled in our design. As a client-owned cohort drawn from a limited geographic area, practical constraints prevented precise matching across these factors while recruiting dogs with severe, naturally occurring CCD.

As with any exploratory, untargeted metabolomics study, this discovery-based approach faces the challenge of accurate metabolite identification, as structural assignments rely on mass spectrometry fragmentation patterns and database matching, which can be ambiguous, particularly for compounds with limited reference spectra or structural isomers. While we employed rigorous analytical methods, including tandem mass spectrometry (MS/MS) and cross-referencing with the Human Metabolome Database (HMDB), the possibility of misidentification or detection of closely related structural analogs cannot be entirely excluded for certain metabolites, particularly those without extensive prior characterization in canine plasma. This limitation underscores the importance of confirmatory studies using targeted metabolomics with authenticated chemical standards to validate the identity and abundance of candidate biomarkers. Despite the limitations mentioned above, the overall metabolic signatures identified, particularly those involving well-characterized lipid classes and oxidative stress markers, demonstrate robust internal consistency and strong alignment with established AD pathophysiology, lending confidence to the biological validity of our findings.

Cognitive assessment in this study relied exclusively on the Canine Dementia Scale (CADES), which enabled clear classification of severely affected dogs (CADES ≥45) versus cognitively normal controls (CADES ≤7). We acknowledge that additional instruments, such as the Canine Cognitive Dysfunction Rating scale (CCDR) or the domain-specific DISHA framework (Disorientation, altered social Interaction, Sleep–wake cycle disturbance, House-soiling, Activity changes), could have provided complementary phenotypic information. Accordingly, we were unable to explore correlations between individual behavioral domains and specific metabolite profiles. Future studies should incorporate multi-scale cognitive assessment to determine whether identified metabolic signatures reflect global cognitive impairment or specific neurobehavioral phenotypes.

A defining strength of this study is the use of community-dwelling companion dogs with naturally occurring CCD, a model system that is unparalleled in its translational fidelity to human Alzheimer’s disease (McGrath et al., 2025). Unlike laboratory animals with experimentally induced pathology housed in controlled facilities, companion dogs cohabit the human household and share the complete exposome of their owners, including environmental pollutants, microbiome influences, psychosocial stressors, sleep–wake cycles, dietary patterns, physical activity levels, and other lifestyle factors that fundamentally shape aging trajectories and disease susceptibility. This shared microenvironment is a unique feature that distinguishes companion dogs from other animal models in neurodegenerative research. No other species develops spontaneous AD-like pathology while simultaneously experiencing the complex, real-world environmental exposures that characterize human aging. This ecological validity substantially enhances the translational relevance of our findings, as the metabolic alterations we observe emerge within the same environmental context that influences human dementia risk. Moreover, the genetic diversity of companion dog breeds more closely mirrors human population heterogeneity than that of inbred laboratory strains, further strengthening the biological relevance of this model. The study of client-owned dogs with naturally occurring neurodegenerative disease, therefore, offers a critical bridge between highly controlled but artifactual laboratory models and the heterogeneous, environmentally embedded reality of human Alzheimer’s disease.

## 5 Conclusion

Metabolomic profiling of aged dogs with CCD reveals systemic metabolic disturbances, particularly in lipid metabolism, mitochondrial function, and oxidative stress that closely resemble those observed in humans with AD. These findings reinforce the translational value of client-owned companion dogs as a spontaneous animal model for AD research. Unlike traditional laboratory models that involve purpose-bred Beagles, companion dogs exhibit great genetic diversity and live in home environments that reflect real-world exposures, making them uniquely suited for studying the complex interplay among environmental exposures, aging, and neurodegeneration. Prospect studies with larger, longitudinal cohorts are essential to validate these candidate biomarkers and clarify their temporal association with cognitive decline. Integrating metabolomic data with neuroimaging, histopathology, and behavioral assessments will be critical to strengthening the mechanistic understanding and translational potential of this model in AD research. Continued exploration of these pathways holds promise for advancing translational efforts of relevance to effective management through novel disease-modifying therapies for Alzheimer’s disease.

Precedent for this strategy is demonstrated by the success of the National Cancer Institute’s Comparative Oncology Program and the Comparative Oncology Trials Consortium, which study naturally occurring cancers in companion dogs as a model system for human disease (National Cancer Institute, Center for Cancer Research, 2019). Senescent companion dogs with CCD could similarly support comparative gerontology and AD-focused research within a National Institute on Aging framework.

## Data Availability

The original contributions presented in the study are included in the article/Supplementary material, further inquiries can be directed to the corresponding author.
