# Supplementary material for: Untargeted plasma metabolomics in canine cognitive dysfunction: the naturally occurring Alzheimer’s disease analog in dogs
Source: Front Neurosci. 2026 Mar 17;20:1681817. doi: 10.3389/fnins.2026.1681817 (PMC13036105; doi:10.3389/fnins.2026.1681817)
Supplement: Supplementary file 1 [file Data_Sheet_1.PDF]

Figure S1

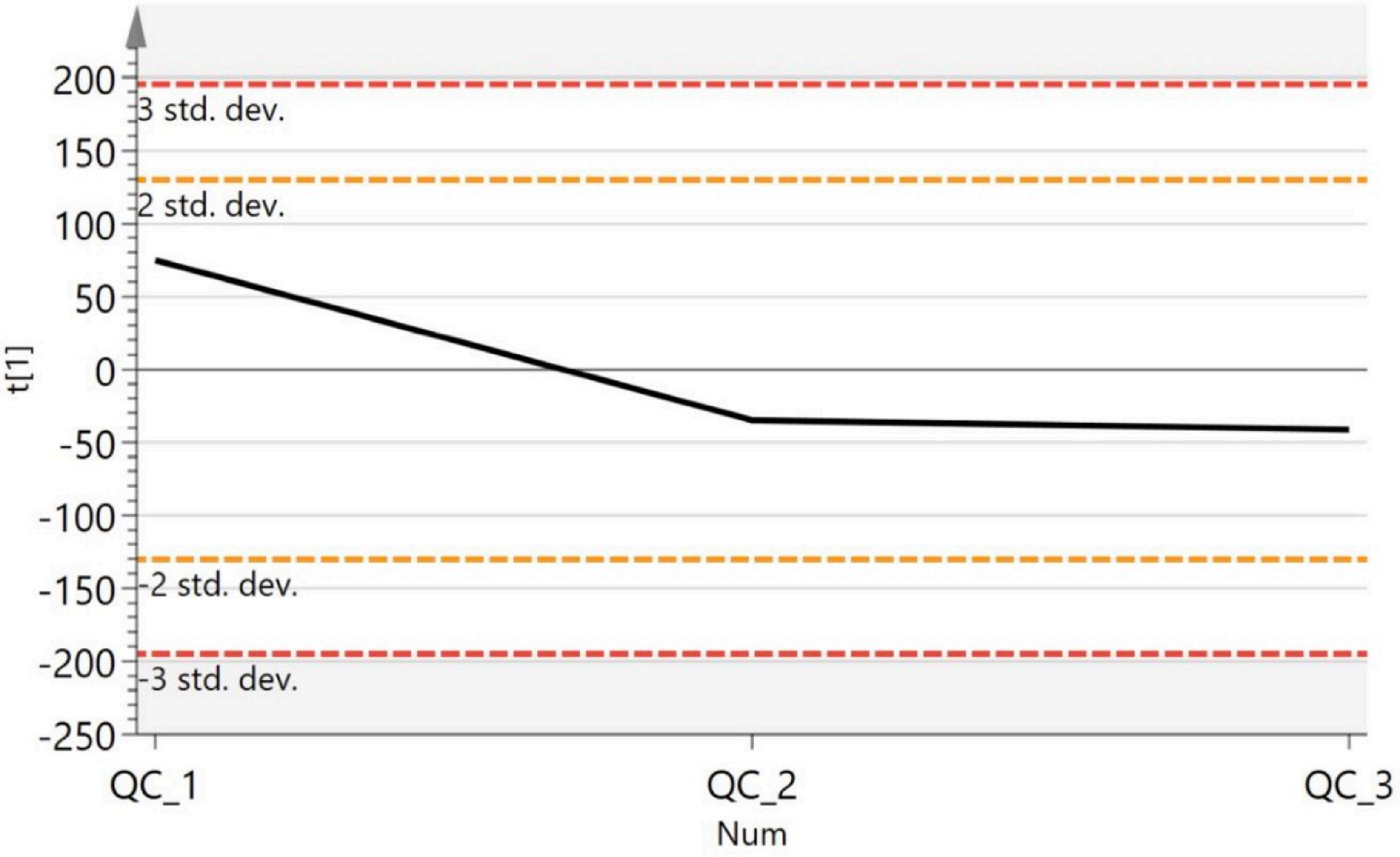

200
150
100
50
0
-50
-100
-150
-200
-250
t[1]
3 std. dev.
2 std. dev.
-2 std. dev.
-3 std. dev.
QC_1
QC_2
QC_3
Num

Figure S1. Quality control assessment of LC-MS system performance. To monitor analytical stability throughout the metabolomics run, pooled quality control (QC) samples were injected at regular intervals in both positive and negative ionization modes. The distribution of relative standard deviation (RSD) values across detected features is shown. Most features exhibited RSDs below 30%, indicating high reproducibility and robustness of the LC-MS platform used in this study.
