# Supplementary material for: Untargeted plasma metabolomics in canine cognitive dysfunction: the naturally occurring Alzheimer’s disease analog in dogs
Source: Front Neurosci. 2026 Mar 17;20:1681817. doi: 10.3389/fnins.2026.1681817 (PMC13036105; doi:10.3389/fnins.2026.1681817)
Supplement: Supplementary file 2 [file Table_1.DOCX]

**Table S1. Signalment of canine study participants**

| **Dog ID** | **Age (yr)** | **Dog Breed(s)** | **Gender** | **Weight (kg)** | **CADES score** | **Group** |
| --- | --- | --- | --- | --- | --- | --- |
| 1 | 11 | Dachshund | MN | 9.6 | 79 | CCD |
| 2 | 16 | Lab mix | MN | 18.8 | 64 | CCD |
| 3 | 14 | Chihuahua mix | MN | 3.7 | 86 | CCD |
| 4 | 17 | Chihuahua | FS | 2.8 | 81 | CCD |
| 5 | 13 | Australian Shepherd | FS | 18.0 | 74 | CCD |
| 6 | 14 | Chihuahua | FS | 7.8 | 6 | CH |
| 7 | 13 | Chihuahua | FS | 3.3 | 6 | CH |
| 8 | 10 | Lab mix | FS | 34.5 | 4 | CH |
| 9 | 10 | German Pointer | FS | 31.0 | 2 | CH |
| 10 | 10 | Boxer | MN | 38.2 | 4 | CH |

CCD: canine cognitive dysfunction; CH: clinically healthy; FS: female spayed; MN: male neutered
