# Supplementary material for: Untargeted plasma metabolomics in canine cognitive dysfunction: the naturally occurring Alzheimer’s disease analog in dogs
Source: Front Neurosci. 2026 Mar 17;20:1681817. doi: 10.3389/fnins.2026.1681817 (PMC13036105; doi:10.3389/fnins.2026.1681817)
Supplement: Supplementary file 3 [file Table_2.DOCX]

**Table S2. Mean canine dementia scale (CADES) scores in dogs with canine cognitive dysfunction (CCD) vs controls**

|  | **CCD** | **Controls** | **p-value** |
| --- | --- | --- | --- |
| **A. Spatial orientation** |  |  |  |
| 1. Disorientation in a previously familiar environment (inside/outside) |  |  |  |
| 1. Unable to recognize previously familiar people or other animals (inside/outside) |  |  |  |
| 1. Abnormal response to previously familiar objects (e.g., furniture, household objects) |  |  |  |
| 1. Aimless wandering (restless) |  |  |  |
| 1. Reduced ability to do a previously learned task |  |  |  |
| Score* [0-25] | 18.8 ± 3.3  (14 – 23) | 0.4 ± 0.9  (0 – 2) | 0.0001 |
| **B. Social interaction** |  |  |  |
| 1. Changes in interactions with people and other dogs (playing, petting, welcoming, etc.) |  |  |  |
| 1. Changes in the dog’s individual behaviors (exploration, play, performance, etc.) |  |  |  |
| 1. Abnormal response to commands and inability to learn new tasks |  |  |  |
| 1. Irritability |  |  |  |
| 1. Aggression |  |  |  |
| Score* [0-25] | 19.4 ± 1.1  (18 – 21) | 3.6 ± 0.9  (2 – 4) | < 0.0001 |
| **C. Sleep-wake cycles** |  |  |  |
| 1. Abnormal nocturnal behaviors (wandering, vocalization, restless, etc.) |  |  |  |
| 1. Switch from insomnia to hypersomnia |  |  |  |
| 2 x Score* [0-20] | 16.0 ± 3.5  (10 – 18) | 0 ± 0  (N/A) | 0.0005 |
| **D. House soiling** |  |  |  |
| 1. Eliminates indoors in random locations |  |  |  |
| 1. Eliminates in kennel and/or sleeping area |  |  |  |
| 1. Changes in signaling behavior prior to elimination activity |  |  |  |
| 1. Eliminates indoors after a recent walk outside |  |  |  |
| 1. Eliminates at uncommon locations (grass, concrete, etc.) |  |  |  |
| Score* [0-25] | 22.6 ± 1.9  (20 – 25) | 0.4 ± 0.9  (0 – 2) | < 0.0001 |
| **Total Score** | 76.8 ± 8.3  (64 – 86) | 4.4 ± 1.7  (2 – 6) | < 0.0001 |

* Canine dementia scale (CADES) frequency: 0 points – abnormal behavior of the dog was never observed, 1 point - abnormal behavior of the dog may have been observed, 2 points – abnormal behavior of the dog was detected at least once in the last 6 months, 3 points – abnormal behavior appeared at least once per month, 4 points – abnormal behavior was seen 2–4 times per month, 5 points – abnormal behavior was observed several times a week. Total score = A + B + C + D (0–95). Clinical stage: Normal aging (0-7). Mild cognitive impairment (8-23). Moderate cognitive impairment (24-44). Severe cognitive impairment (45-95). Based on Madari A, *et al.* Applied Animal Behaviour Science 171 (2015), 138-145 (modified wording). The table shows mean ± standard deviation (min - max) for client-owned companion dogs with canine cognitive dysfunction (CCD) compared to clinically healthy (CH) controls.
