# Supplementary material for: Untargeted plasma metabolomics in canine cognitive dysfunction: the naturally occurring Alzheimer’s disease analog in dogs
Source: Front Neurosci. 2026 Mar 17;20:1681817. doi: 10.3389/fnins.2026.1681817 (PMC13036105; doi:10.3389/fnins.2026.1681817)
Supplement: Supplementary file 4 [file Table_3.DOCX]

**Table S3. Hematological and serum biochemical profiles of dogs with canine cognitive dysfunction (CCD) vs controls**

| **Complete Blood Count (CBC)** | | | | | | | | | |
| --- | --- | --- | --- | --- | --- | --- | --- | --- | --- |
|  | **CCD** | **Controls** | **p-value** | **Reference** |  | **CCD** | **Controls** | **p-value** | **Reference** |
| Erythrocytes (M/μL) | 6.52 ± 0.73  (5.25 - 7.15) | 7.27 ± 1.26  (5.85 - 9.03) | 0.29 | 5.65 - 8.87 | Lymphocytes (K/μL) | 1.67 ± 0.77  (0.96 - 2.83) | 1.83 ± 0.44  (1.32 - 2.30) | 0.70 | 1.05 - 5.10 |
| Hematocrit (%) | 40.0 ± 3.8  (33.4 - 43.3) | 47.7 ± 7.7  (39.1- 56.3) | 0.09 | 37.3 - 61.7 | Monocytes (K/μL) | 0.76 ± 0.49  (0.41 - 1.60) | 0.51 ± 0.05  (0.43 - 0.55) | 0.32 | 0.16 - 1.12 |
| Hemoglobin (g/dL) | 14.9 ± 1.5  (12.3 - 16.3) | 16.8 ± 3.0  (13.3 - 20.3) | 0.24 | 13.1 - 20.5 | Eosinophil (K/μL) | 0.67 ± 0.78  (0.24 - 2.07) | 0.31 ± 0.14  (0.09 - 0.44) | 0.35 | 0.06 - 1.23 |
| Leukocytes (K/μL) | 12.18 ± 8.68  (7.18 - 27.29) | 11.18 ± 4.11  (8.48 – 18.40) | 0.82 | 5.05 - 16.76 | Basophil (K/μL) | 0.05 ± 0.05  (0 - 0.11) | 0.04 ± 0.02  (0.02 - 0.07) | 0.72 | 0.00 - 0.10 |
| Neutrophils (K/μL) | 9.03 ± 7.57  (4.73 - 22.46) | 8.49 ± 3.86  (5.81 - 15.32) | 0.89 | 2.95 - 11.64 | Platelets (K/μL) | 415 ± 239  (117 - 763) | 345 ± 123  (185 - 524) | 0.58 | 148 - 484 |
| **Serum Chemistry Panel (SCP)** | | | | | | | | | |
|  | **CCD** | **Controls** | **p-value** | **Reference** |  | **CCD** | **Controls** | **p-value** | **Reference** |
| Glucose (mg/dL) | 104 ± 13  (85 - 120 | 110 ± 17  (88 - 134) | 0.55 | 74 - 143 | Chloride (mmol/L) | 115 ± 3  (112 - 118) | 113 ± 4  (108 - 118) | 0.45 | 109 - 122 |
| Creatinine (mg/dL) | 0.9 ± 0.4  (0.6 - 1.6) | 0.8 ± 0.2  (0.5 - 1.1) | 0.71 | 0.5 - 1.8 | Total Protein (g/dL) | 7.1 ± 0.3  (6.8 - 7.6) | 7.0 ± 0.4  (6.5 - 7.6) | 0.49 | 5.2 - 8.2 |
| BUN (mg/dL) | 22 ± 11  (11 - 40) | 16 ± 6  (9 - 25) | 0.27 | 7 - 27 | Albumin (g/dL) | 3.4 ± 0.3  (3.0 - 3.7) | 3.4 ± 0.4  (3.0 - 4.1) | 0.93 | 2.3 - 4.0 |
| Phosphorus (mg/dL) | 4.4 ± 1.0  (3.0 - 5.9) | 4.5 ± 1.2  (3.2 - 6.1) | 0.91 | 2.5 - 6.8 | ALT (U/L) | 74 ± 41  (31 - 127) | 48 ± 13  (27 - 60) | 0.24 | 10 - 125 |
| Calcium (mg/dL) | 10.3 ± 0.2  (10.1 - 10.7) | 9.9 ± 0.7  (9.3 - 11.0) | 0.32 | 7.9 - 12.0 | ALP (U/L) | 245 ± 174  (62 - 460) | 195 ± 157  (48 - 366) | 0.64 | 23 - 212 |
| Sodium (mmol/L) | 150 ± 2  (147 - 153) | 147 ± 1  (146 - 149) | 0.08 | 144 - 160 | GGT (U/L) | 0.6 ± 0.9  (0 - 2) | 0.6 ± 0.5  (0 - 1) | 1.00 | 0 - 11 |
| Potassium (mmol/L) | 5.0 ± 0.6  (4.3 – 5.8) | 4.5 ± 0.4  (4.2 – 5.0) | 0.20 | 3.5 - 5.8 | Bilirubin (mg/dL) | 0.2 ± 0.1  (0.1 - 0.4) | 0.3 ± 0.2  (0.1 – 0.6) | 0.56 | 0.0 - 0.9 |

The CBC and SCP analyses were performed at the WesternU Pet Health Center using a ProCyte Dx Hematology Analyzer (CBC), and Catalyst One Chemistry Analyzer (serum chemistry), both from IDEXX Laboratories (Westbrook, ME). Data was analyzed using MS Excel for Mac (Version 16.97.2) for descriptive and inferential statistics (t-test and alpha level of 0.05). Values reflect mean ± std (min – max) for dogs with canine cognitive dysfunction compared to controls. Reference values were provided by IDEXX.
